# Child and adolescent psychiatry consultations during the COVID-19 pandemic

**DOI:** 10.1038/s41598-025-08566-2

**Published:** 2025-07-01

**Authors:** Zeynep Vatansever Pınar, İrem Damla Çimen, Elif Küçük, Mehmet Tolga Köle, İbrahim Kandemir

**Affiliations:** 1 Department of Child and Adolescent Psychiatry, Maltepe University School of Medicine, Istanbul, Turkey; 2 Department of Child and Adolescent Psychiatry, Kocaeli University School of Medicine, Kocaeli, Turkey; 3 Department of Psychiatry, Yüksek İhtisas School of Medicine, Ankara, Turkey; 4 Department of Pediatrics, Ankara Etlik City Hospital, Ankara, Turkey; 5 Department of Pediatrics, Istanbul Health and Technology University, Istanbul, Turkey

**Keywords:** COVID-19, Pandemic, Consultation liaison psychiatry, Child and adolescent, Face-to-face education, Suicide attempt, Human behaviour, Quality of life

## Abstract

This study aimed to identify the biopsychosocial stressors influencing child and adolescent mental health during periods when facetoface education was suspended and to evaluate the psychological sequelae of school closures and related restrictions. We conducted a retrospective observational study at Kartal Dr. Lütfi Kırdar City Hospital, a tertiary care center in İstanbul, reviewing all patients referred to the child and adolescent consultation-liaison psychiatry (CLP) unit between March 2020 and March 2022. We assessed associations between psychiatric diagnoses and health-related stressors, school closure status, age, and sex using both frequentist and Bayesian methods. During the study period, 264,013 pediatric admissions were recorded, of whom 270 (0.10%) required psychiatric consultation. The proportions of suicide attempts and anxiety diagnoses did not differ between periods of open and closed schools (BF10 = 0.21 and 0.138, respectively; moderate evidence for the null). Multivariate analysis showed that the odds of suicide attempts were higher in patients exposed to family or schoolrelated stressors (OR = 6.63, 95% CI 2.72–16.19), in females (OR = 8.10, 95% CI 4.16–15.77), and with increasing age (OR = 1.32 per year, 95% CI 1.16–1.50). Female sex (OR = 4.17, 95% CI 2.03–8.55) and older age (OR = 1.30 per year, 95% CI 1.12–1.50) were also associated with depression. Anxiety was more likely in those facing isolation or healthrelated stressors (OR = 3.91, 95% CI 1.66–9.22). These findings highlight the differential impact of stressor type on internalizing symptoms and may inform resource allocation and crisisresponse planning in child mental health services.

## Introduction

The coronavirus disease 2019 (COVID-19) pandemic, first identified in Wuhan, China, rapidly spread worldwide and generated profound distress among children, adults, healthcare personnel, and the general population^1–3^. Measures designed to limit viral transmission fear of infection, social isolation, and quarantine—precipitated psychosocial difficulties and adversely affected mental health, particularly in individuals without prior experience of large-scale crises^4^. In March 2020, the World Health Organization declared COVID-19 a global pandemic^5^. Since then, the pandemic has unfolded in successive waves worldwide, placing sustained pressure on healthcare systems for more than two years. The prevalence and severity of these waves have differed among countries, reflecting variations in public health policies and vaccination programs, as evidenced in Europe, Canada, and Iran^6,7^.

In Turkey, schools were closed on 23 March 2020, and remote online education commenced; a curfew for individuals under 20 years of age followed on 4 April 2020, further limiting children’s mobility. Although all age groups were affected by the pandemic, children and adolescents were especially vulnerable owing to the disruption of routines and the absence of school-based coping mechanisms^8^. Prolonged time at home has been reported to confer both potential benefits (e.g., respite from bullying or academic strain) and risks (e.g., family conflict or exposure to violence)^9,10^.

Child and adolescent consultation liaison psychiatry (CLP) provides a multidisciplinary, biopsychosocial framework adopted in the evaluation and treatment field of young patients with co-occurring physical illness and behavioral-emotional difficulties, offering a holistic approach to their comprehensive care. The increased psychiatric burden observed during the pandemic underscored the importance of hospital-based CLP services^11^. In the tertiary center where the present study was conducted, pediatricians or other specialists submit electronic referrals when psychiatric symptoms are observed in hospitalized or emergency department patients, after which child psychiatrists assign the Diagnostic and Statistical Manual of Mental Disorders, Fifth Edition (DSM-5) diagnoses and formulate intervention plans.

In this study, a consultation request denotes every formal referral logged in the hospital’s electronic system, irrespective of the case’s duration or complexity. Defining this term is critical, as referral pathways and terminology can differ across institutions and healthcare systems. Because isolation measures during the COVID-19 pandemic restricted access to outpatient care, we limited our analysis to CLP services delivered to children and adolescents hospitalized or evaluated in the emergency department. Accordingly, we addressed the following research question:

To what extent are depression, anxiety, and suicidal behavior predicted by age, sex, psychosocial stressor type (health-related vs. non-health-related), and school closure status during the pandemic?

A secondary objective was to identifying areas for interprofessional collaboration and task allocation within child and adolescent CLP, thereby informing strategies to strengthen service delivery in future public health crises.

## Materials and methods

The study was approved by the Ministry of Health Ethics Committee on 23 February 2022 and by Kartal Dr. Lütfi Kırdar City Hospital Medical Research Ethics Committee on 9 March 2022 (Decision No. 2022/514/221/8). All procedures conformed to the Declaration of Helsinki.

### Participants and eligibility criteria

This retrospective observational study comprised all children and adolescents (0–18 years) referred to the Child and Adolescent Consultation‑Liaison Psychiatry (CLP) service of Kartal Dr. Lütfi Kırdar City Hospital—a tertiary-care center in İstanbul—between March 2020 and March 2022. Patient data were obtained from electronic medical records.

**Inclusion criteria**:

- Age between 0 and 18 years,

- Referral for CLP evaluation during an inpatient admission (including intensive care and general pediatrics) or while under assessment in the emergency department.

**Exclusion criteria**:

- Incomplete or missing clinical data,

- Age > 18 years of referral.

All cases satisfying the inclusion criteria and none of the exclusion criteria were retained as the final analytic sample. All available data for patients under the age of 18 were included in the study.

Before conducting the analyses, all psychiatric consultation records were reviewed for completeness. An entry was considered incomplete if any of the following key variables were missing: (1) age or sex; (2) primary psychiatric diagnosis; (3) type of reported stressor (health-related or non-health-related); or (4) consultation outcome (e.g., admission, discharge). Entries missing one or more of these variables were planned to be excluded from the dataset to maintain data integrity. However, all consultation records from the defined study period were complete for these variables, and thus no cases were excluded due to missing data.

Kartal Dr. Lütfi Kırdar City Hospital is a 1,105-bed tertiary-care center that provides advanced emergency, outpatient, and inpatient services to a large catchment population. The facility includes 92 pediatric beds and a 24-bed pediatric intensive-care unit.

The analysis encompassed sociodemographic variables, the department submitting the consultation request, the reason for referral, the patient’s psychiatric diagnosis, and identified stressors. Child and adolescent psychiatrists assigned diagnoses according to DSM-5 criteria during routine consultation-liaison assessments. Diagnostic decisions were informed by psychiatric interviews with patients and caregivers, mental status examinations, and the review of pertinent medical records.

### Identification of consultation reason

The indication for each consultation was identified by examining the referring clinician’s note alongside the accompanying psychiatric response.

### Identification and classification of stressors

Psychosocial stressors were elicited during routine consultation-liaison interviews conducted by child and adolescent psychiatrists. These interviews explored the child’s recent difficulties, presumed sources of distress, and contextual factors—school, peer relationships, family dynamics, health concerns, or pandemic-related isolation. Stressors were not coded with a standardized instrument; rather, clinicians extracted recurring themes during data collection and subsequently classified them into two broad groups: health-related (e.g., illness, quarantine) and non-health-related (e.g., family conflict, academic pressure, school closures). In cases where both types of stressors were reported, classification was based on the primary stressor emphasized during the consultation, as documented in the clinical notes.

### Classification of suicide attempt and non-suicidal self-injury (NSSI)

In this study, suicide attempts and non-suicidal self-injury (NSSI) were coded as separate phenomena. NSSI was defined as deliberate, self-inflicted harm carried out without intent to die. Behavior was classified as a suicide attempt only when explicit suicidal intent was evident—either from the patient’s report or from clinical documentation^12^.

### Statistical analysis

Categorical variables were presented as frequencies (n) and percentages (%). Comparisons were made using the chi-squared test, and correlations with Kendall’s tau test. We also built multivariate models using binomial logistic regression. *p* < 0.05 was accepted as the significance level.

Also, we built H0 (independence) and H1 (correlation) hypothesis and subjected the data into Bayesian calculations and presented the evidence via Bayes factors. BF10 results between 1 and 3 mentions anecdotal evidence, between 3 and 10 moderate evidence, 10–30 strong evidence, 30–100 very strong evidence, and > 100 extreme evidence for H1(correlation hypothesis). BF10 results between 1/3 and 1 mentions anecdotal evidence for that there is no difference (H0 hypothesis), 1/3 and 1/10 moderate evidence, 1/10 and 1/30 strong evidence, 1/30 and 1/100 very strong evidence. We set stretched beta prior width at 1.

All calculations were performed with SPSS version 26 and Jamovi 2.3.18 statistical package programs.

## Results

**Fig. 1:**
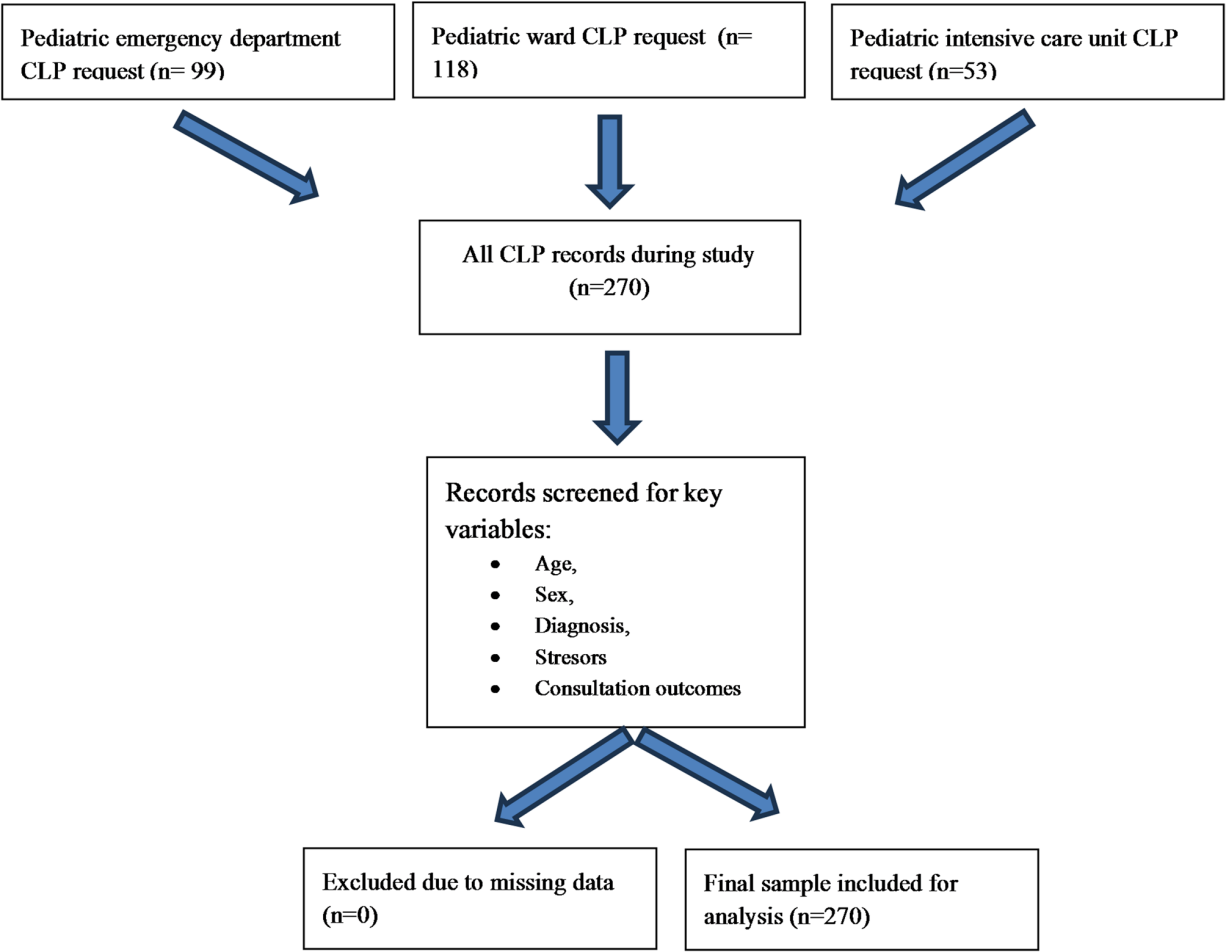
Flow Diagram.

During the two-year span that encompassed the onset, peak, and restriction phases of the COVID-19 pandemic, 256,931 children presented to the pediatric emergency department of Kartal Dr. Lütfi Kırdar City Hospital, and 7,082 were admitted to pediatric wards. CLP input was requested for 0.03% of emergency presentations, 1.67% of pediatric inpatients, and 5.6% of patients in the pediatric intensive-care unit (Fig. 1). Taken together, psychiatric consultation was sought for 0.1% of all pediatric encounters (outpatient and inpatient combined). The analytic sample comprised 270 patients—180 females (66.6%) and 90 males (33.3%)—with a mean age of 14.7 ± 2.7 years for females, 13.6 ± 3.9 years for males, and 14.4 ± 3.2 years overall. Overall, 17.4% of patients were younger than 12 years, whereas 82.6% were aged 12 years or older. The number of consultation requests per patient ranged from 1 to 6, with a mean ± SD of 1.36 ± 0.83.

The leading indications for consultation were suicide attempts, general psychiatric assessment, and agitation/fear/crying. While 64.4% of the patients had a psychiatric diagnosis, 35.6% had no psychiatric diagnosis. Among those diagnosed, the three most prevalent conditions were depressive disorder, anxiety disorder, and adjustment disorder (Table 1).

**Table 1:** Primary reasons for consultation requests and clinical diagnoses by sex, along with age distribution of the sample.

		Female	Male	Total
		Mean ± Sd	Mean ± Sd	Mean ± Sd
**Age**		14.7 ± 2.7	13.6 ± 3.9	14.4 ± 3.2

		Female	Male	Total
		n (%)	n (%)	n (%)
**Sex**		180 (66,6)	90 (33,3)	270 (100)
**Diagnoses**	Depressive disorder	69 (25,5)	11 (4,1)	80 (29,6)
	Anxiety disorder	16 (5,9)	9 (3,4)	25 (9,3)
	Adjustment disorder	7 (2,6)	11 (4,1)	18 (6,7)
	Conduct disorder	9 (3,3)	2 (0,7)	11 (4,1)
	Delirium	2 (0,7)	5 (1,9)	7 (2,6)
	Dissociative Disorders	4 (1,5)	2 (0,7)	6 (2,2)
	Acute stress disorder	3 (1,1)	3 (1,1)	6 (2,2)
	ADHD	0 (0,0)	4 (1,5)	4 (1,5)
	Substance use disorder	0 (0,0)	3 (1,1)	3 (1,1)
	Eating disorder	2 (0,7)	0 (0,0)	2 (0,7)
	Bipolar disorder	2 (0,7)	0 (0,0)	2 (0,7)
	Dystonia	0 (0,0)	2 (0,7)	2 (0,7)
	Mental retardation	2 (0,7)	0 (0,0)	2 (0,7)
	OCD	2 (0,7)	0 (0,0)	2 (0,7)
	Other	0 (0,0)	4 (1,5)	4 (1,5)
	Not diagnosed	62 (23,0)	34 (12,6)	96 (35,6)
**Reasons for requesting consultations**	Suicide attempt	124 (45,9)	21 (7,8)	145 (53,7)
	Psychiatric assessment	16 (5,9)	21 (7,8)	37 (13,7)
	Agitation, fear and crying	10 (3,7)	15 (5,6)	25 (9,3)
	Depressive symptoms	6 (2,2)	6 (2,2)	12 (4,4)
	Non-compliance with the disease or treatment	3 (1,1)	7 (2,6)	10 (3,7)
	Auditory-visual hallucinations	4 (1,5)	4 (1,5)	8 (3,0)
	Behavior problems	5 (1,9)	3 (1,1)	8 (3,0)
	Somatic complaints	6 (2,2)	2 (0,7)	8 (3,0)
	Contractions	2 (0,7)	5 (1,9)	7 (2,6)
	Preop evaluation	0 (0,0)	5 (1,9)	5 (1,9)
	Other	4 (1,3)	1 (0,3)	5 (1,7)

ADHD: Attention deficit hyperactivity disorder, OCD: Obsessive-compulsive disorder.

Regarding psychosocial stressors, 40.7% of patients (*n* = 110) reported school-, peer-, or friendship-related difficulties; 40.0% (*n* = 108) experienced family conflict, bereavement, or similar issues; and 19.3% (*n* = 52) faced isolation or health-related concerns.

Consultations were more frequent during school closures (67.4%, *n* = 182) than when schools were open (32.6%, *n* = 88). Notably, referrals for suicide attempts comprised 34.4% of consultations during closures (*n* = 93) compared with 19.3% when schools were in session (*n* = 52).

NSSI was documented in 13% of patients (*n* = 35), whereas suicide attempts were recorded in 53% (*n* = 143). The predominant method was medication ingestion (90.9%, *n* = 130), followed by jumping from height (5.6%, *n* = 8), toxic ingestion (2.1%, *n* = 3), hanging (0.7%, *n* = 1), and use of a weapon (0.7%, *n* = 1). Patients reported between one and six attempts, with 82.5% representing first-time episodes (Table 2).

**Table 2:** Distribution of suicide attempt status and types according to sex.

		Female	Male	Total	*p*
		*n* (%)	*n* (%)	*n* (%)	
Suicide attempt	Yes	124(45,9)	19(7)	143(52,9)	0,001^*^
	No	56(20,7)	71(26,3)	127(47)	
Suicide attempt types	Medicine	114(79,7)	16(11,2)	130(90,9)	0,66
	Jumping from height	6 (4,2)	2(1,4)	8(5,6)	
	Toxic substance	2(1,4)	1(0,7)	3(2,1)	
	Hanging	1(0,7)	0(0)	1(0,7)	
	weapon	1(0,7)	0(0)	1(0,7)	

*p < 0,05, chi-square test.

Multivariate analyses incorporating age, sex, stressor type (health-related vs. non-health-related), and school status (open vs. closed) revealed that age, sex, and stressor type were all significantly associated with both suicide attempts and depression. Anxiety, by contrast, was linked only to stressor type.

School status (open vs. closed) was not associated with the prevalence of suicide attempts, depression, or anxiety. Bayesian analyses provided moderate evidence for no difference in suicide attempts (BF10 = 0.21) and anxiety (BF10 = 0.138) between school-status groups, and only anecdotal evidence for no difference in depression (BF10 = 0.388) (Bayesian Kendall’s tau test) (Table 3).

**Table 3:** Correlation co-efficient between suicide attempt, depression, anxiety with age, school closure status, stressor types, and sex.

	Suicide Attempt		Depression		Anxiety	
	r	*p*	r	*p*	r	*p*
Age	0.224	**< 0.001**	0.162	**< 0.001**	-0.037	0.464
School closure status	0.065	0.445	0.091	0.285	-0.038	0.652
Stressor Types	0.368	**< 0.001**	0.173	**0.005**	-0.2	**< 0.001**
Sex	-0.451	**< 0.001**	-0.27	**< 0.001**	0.018	0.767

Kendall’s tau correlation coefficients (τ) and corresponding p-values are presented.

Negative correlations with sex indicate higher rates in females (sex coded as 0 = male, 1 = female).

“Stressor types” refers to the nature or severity of the reported psychosocial stressor (e.g., isolation vs. family/school-related issues).

Statistically significant results are shown in bold. *p < 0,*05.*

**Fig. 2:**
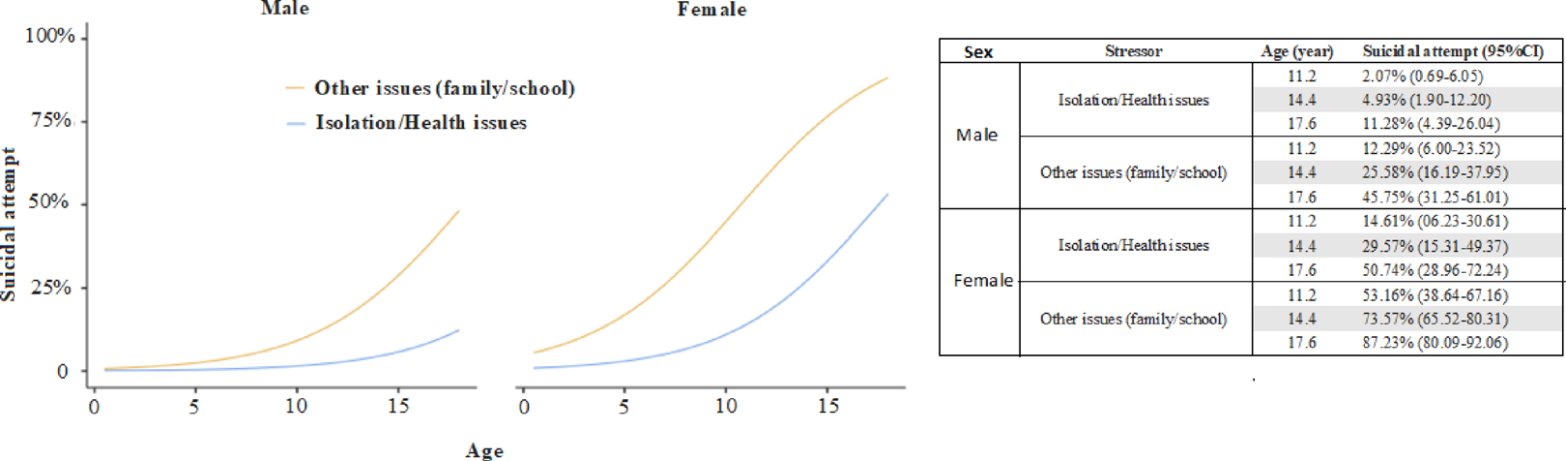
The multivariate analysis figure regarding suicide attempt and sex, stressor type, and age (binomial logistic regression).

In the multivariate model, exposure to non-health-related stressors (family or school) increased the odds of a suicide attempt 6.63-fold (95% CI 2.72–16.19) relative to isolation/health-related stressors, while female sex raised the odds 8.10-fold (95% CI 4.16–15.77). Each additional year of age was associated with a 32% increase in risk (OR = 1.32, 95% CI 1.16–1.50) (R_2_McF:0.299) (Fig. 2**).**

**Fig. 3:**
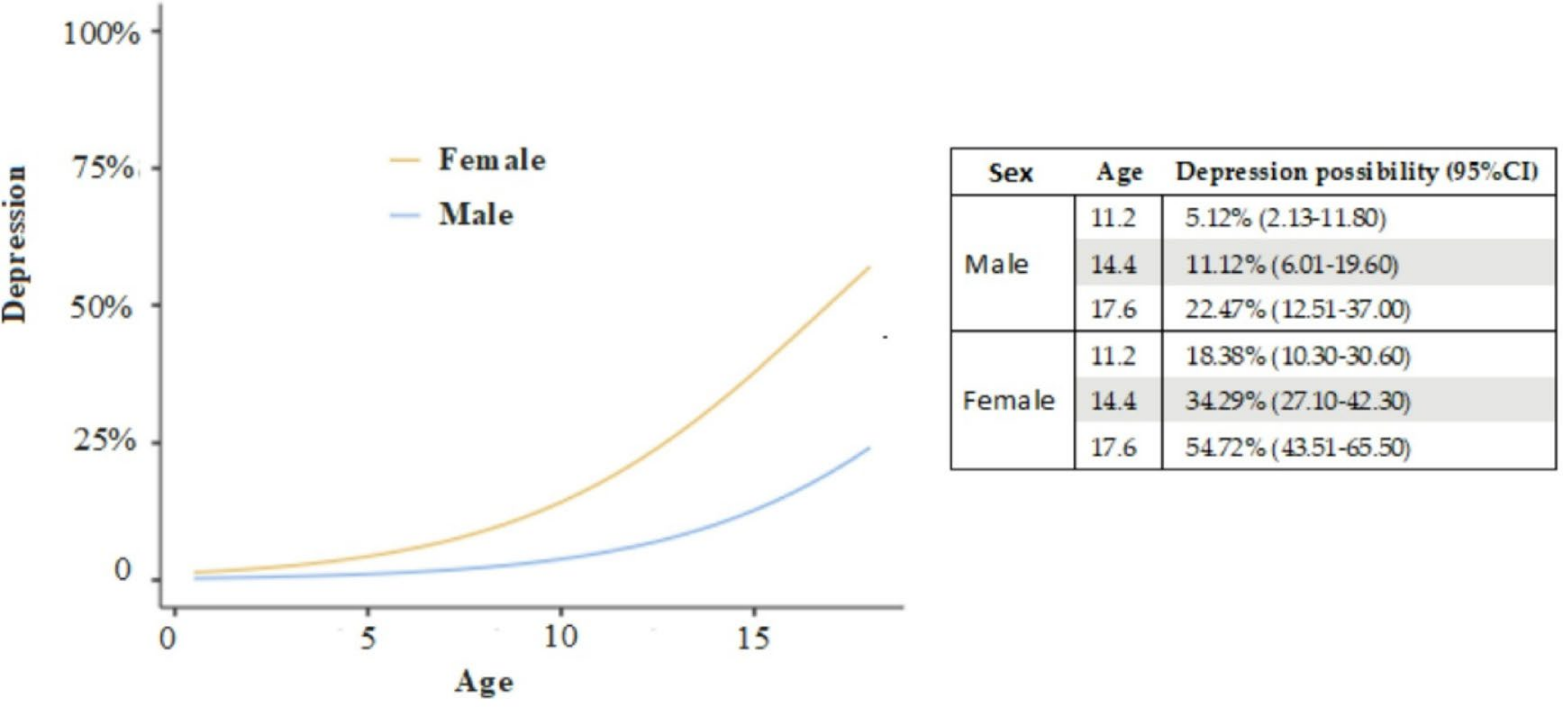
The multivariate analysis figure regarding depression and sex, and age (binomial logistic regression).

In the multivariable model, female participants had 4.17-fold higher odds of receiving a depression diagnosis than male participants (OR = 4.17; 95% CI 2.03–8.55), and each additional year of age was associated with a 1.30-fold increase in odds (OR = 1.30 per year; 95% CI 1.12–1.50) (R_2_McF:0.121) (Fig. 3).

**Fig. 4:**
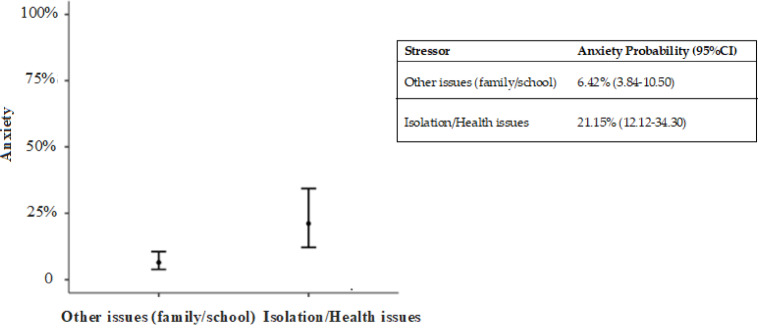
The multivariate analysis figure regarding anxiety and stressor types (binomial logistic regression).

For anxiety, the multivariable model indicated that exposure to isolation- or health-related stressors conferred a 3.91-fold increase in the odds of diagnosis compared with family- or school-related stressors (OR = 3.91; 95% CI 1.66–9.22) (R_2_McF:0.05) (Fig. 4).

## Discussion

This study addresses a critical evidence gap by delineating the stressors that undermined child and adolescent mental health during the COVID-19 pandemic and quantifying the psychological impact of prolonged school closures. During school-closure intervals, referrals for suicide-related consultations rose markedly, and female adolescents were significantly over-represented among those requiring suicide-risk assessment and intervention.

In our center, psychiatric consultation was requested for 0.03% of pediatric emergency-department presentations, 1.67% of ward admissions, and 0.10% of all pediatric encounters. By comparison, a U.S. report documented a 31% year-on-year rise in mental-health–related emergency visits among 12- to 17-year-olds in 2020^13^. Prior to the pandemic, the consultation rate for hospitalized pediatric patients was 2.2% over a 12-month period that began two months before COVID-19 emerged^14^. Another study showed that overall consultation rates climbed from 0.55% before the pandemic to 1.32% afterward^15^.

It has been reported that the total number of psychiatric admissions to emergency departments significantly decreased during the pandemic period compared to the previous year^16^. Although population surveys indicate that psychiatric problems among children and adolescents increased overall, our data reveal a different pattern: ward-based consultation rates remained in line with pre-pandemic figures, whereas emergency department referrals fell^17–19^. This trend is consistent with findings from a multicenter analysis that grouped data from ten countries into fourteen regions, reporting uniform declines in child and adolescent psychiatric emergencies during March–April 2020 compared to the same period in 2019, an effect largely attributed to quarantine measures^20^. Potential explanations include reduced help-seeking for mental health concerns, diminished recognition of psychiatric emergencies amid a focus on COVID-19 symptoms, and the redeployment of clinicians to pandemic-related duties. The mean age of our study was 14.4 ± 3.2 years, and adolescents (≥ 12 years) accounted for 82.6% of cases—consistent with pre-pandemic data and with reports that adolescents experienced disproportionate psychological distress during COVID-19, driving a higher demand for consultations in this age group^21–23^.

Our findings are consistent with global evidence demonstrating increased rates of clinically diagnosed depression, anxiety disorder, and suicidal attempts among young people during the COVID-19 pandemic. A meta-analysis estimated that 25.2% of children and adolescents experienced clinically significant depressive symptoms and 20.5% experienced anxiety^18^. In the United States, a CDC survey found that 37.1% of high-school students reported poor mental health and 19.9% had seriously considered suicide attempts in the preceding year^24^. Similarly, an Australian longitudinal study showed that nearly three-quarters of adolescents met clinical thresholds for depression or anxiety by age 18^25^. Collectively, these data indicate that the patterns observed in our Turkish study reflect a broader, worldwide mental-health burden in youth and underscore the need for international cooperation in preventive and early intervention strategies.

Most suicide attempts in our study involved drug ingestion. International evidence likewise shows that self-poisoning is the predominant method among adolescents, both before and during the COVID-19 pandemic. A global multicenter analysis of pediatric emergency data identified intentional ingestion of therapeutic agents—chiefly psychotropics and analgesics—as the leading mechanism, with a pronounced female predominance^26^. A retrospective series spanning 2017–2022 similarly found that nearly all pediatric suicide attempts entailed overdose, with more than half involving multiple substances^27^. Consistent with these reports, 90.9% of suicide attempts in our study were due to oral ingestion of medications.

Jumping from height (5.6%), ingestion of toxic substances (2.1%), hanging (0.7%), and firearm use (0.7%) were far less common; the latter two methods are seldom documented in pediatric literature. The severity and potential lethality of these behaviors may signal the profound psychological distress experienced during the pandemic. Importantly, 82.5% of cases represented a first suicide attempt—a proportion consistent with pre-pandemic reports^28^. This underscores the need for longitudinal psychiatric follow-up, as multiple prior attempts are a well-established predictor of eventual suicide completion.

Our data indicate that school closure status and isolation measures, per se, did not elevate the risk of suicide attempts. A previous study spanning the pre-pandemic and early-pandemic phases identified domestic conflict as the predominant stressor and major depressive disorder as the most frequent diagnosis among youths presenting to emergency departments for suicide attempts^29^; our findings corroborate this pattern. Moreover, family- and school-related stressors further amplified risk in female and adolescent subgroups. Although several reports document pandemic-era increases in suicidal ideation, planning, and attempts^30^, the over-representation of females in our study—coupled with the heightened vulnerability of females to depression during the pandemic—may partly account for the higher volume of suicide-related consultations among females^31,32^. Importantly, suicides occurring during school closures may also be driven by factors unrelated to COVID-19 itself, including prior trauma, personality traits, pre-existing mental health disorders, and previous suicide attempts^9,33^.

Depressive disorder, anxiety disorder, and adjustment disorder were the three most frequent diagnoses in our consultation study—a distribution that mirrors findings from other settings^34^. Depression was diagnosed disproportionately in females, whereas adjustment disorder was more common in males. Supporting this pattern, an increase in depressive and social anxiety symptoms among adolescent females—but a decrease among males—was observed during the pandemic. Collectively, these data indicate that adolescent females carried a heavier mental health burden during COVID-19 and highlight the need for gender-responsive prevention and intervention strategies^35^.

School closure status and isolation measures were not independently associated with a higher likelihood of a depression diagnosis. In line with previous reports, family- and school-related stressors were more frequently reported by female patients^36^. Pandemic-related economic, health, and occupational strains—compounded by supervising children’s remote learning, reduced social support, and financial hardship—substantially heightened parental stress. This heightened stress likely degraded parent–child relationships and fueled intrafamilial conflict and violence^37–39^. The enforced proximity of adolescents to their families during a developmental stage that typically emphasizes autonomy, together with increased parental tension from job loss or remote work and culturally mediated pressures placed more heavily on girls, may have further intensified household discord.

Isolation and school closure measures emerged as the strongest predictors of anxiety in our study. Prior studies likewise show that pandemic restrictions and the suspension of in-person schooling disrupt children’s daily routines and mental health^40^. Parental distress also escalated during closures: depression, anxiety, stress, and trait anger were all significantly higher among parents when schools were shut than when they were open^41^. This heightened parental strain likely strained parent–child relationships and intensified household conflict. For school-aged children, closures meant less physical activity, sharply increased screen time, and the abrupt loss of academic structure. Cancelled examinations and unfamiliar online learning formats further fueled worries about academic performance. Together, these factors probably contributed to the rise in anxiety diagnoses and the growing demand for child and adolescent psychiatric care during the pandemic^42^.

This study has several limitations. First, the absence of pre-pandemic data precluded a full assessment of temporal change. Additional constraints include its retrospective, cross-sectional design; reliance on self-reported information; the absence of structured diagnostic interviews; and a relatively modest sample size. Moreover, stressors were identified and categorized post hoc from clinical notes rather than through a standardized instrument, which may have introduced classification bias. In addition, clinical diagnoses were based on unstructured psychiatric interviews, which may have led to inter-clinician variability over the two-year data collection period. Nonetheless, the study possesses notable strengths: it spans a two-year interval encompassing the height of the pandemic, contrasts periods with and without face-to-face schooling, systematically documents patient-reported stressors, and captures presentations across both emergency and inpatient settings.

## Conclusions

This study delineates the pattern of psychiatric consultations for children and adolescents during the COVID-19 pandemic and shows how presentation rates varied by age, sex, and stressor type. Depression and suicide attempts were strongly associated with female sex and older age, whereas anxiety was most closely linked to health-related or isolation stressors. School closure status, however, had no discernible effect on suicide- or anxiety-related consultations.

These findings highlight the need for structured, crisis-responsive interventions in pediatric consultation-liaison psychiatry. Screening systems capable of detecting internalizing disorders and symptoms—depression, anxiety, and suicidality—should be deployed early among high-risk youth during public health emergencies. Integrating psychosocial support into routine pediatric care is essential for hospitalized patients and those seen in emergency settings. Intervention models must also be sex- and age-responsive, given the heightened vulnerability of older girls to depression and suicidality observed here. Collectively, these insights can inform hospital preparedness and policy development aimed at safeguarding child and adolescent mental health in future crises.

## Data Availability

The data that support the findings of this study are available on request from the corresponding author. The data is not publicly available due to privacy or ethical restrictions.

## References

[1] Vincent, A. et al. Psychological burden in patients with COVID-19 and their relatives 90 days after hospitalization: a prospective observational cohort study. *J. Psychosom. Res.* **147**, 110526 (2021). 34051515 10.1016/j.jpsychores.2021.110526 PMC8132501

[2] Shapiro, P. A. et al. Report of the academy of consultation-liaison psychiatry task force on lessons learned from the COVID-19 pandemic: executive summary. *J. Acad. Consult Liaison Psychiatry*. **62**, 377–386 (2021). 34000470 10.1016/j.jaclp.2021.05.001 PMC8120806

[3] Horn, M., Granon, B., Vaiva, G., Fovet, T. & Amad, A. Role and importance of consultation-liaison psychiatry during the Covid-19 epidemic. *J. Psychosom. Res.* **137**, 110214 (2020). 32798833 10.1016/j.jpsychores.2020.110214 PMC7405828

[4] Duan, L. & Zhu, G. Psychological interventions for people affected by the COVID-19 epidemic. *Lancet Psychiatry*. **7**, 300–302 (2020). 32085840 10.1016/S2215-0366(20)30073-0 PMC7128328

[5] World Health Organization. WHO Director-General’s opening remarks at the media briefing on COVID-19–11 March 2020. Accessed 25. January (2025). https://www.who.int/director-general/speeches/detail/who-director-general-s-opening-remarks-at-the-media-briefing-on-covid-19---11-march-2020

[6] Iftekhar, E. N. et al. A look into the future of the COVID-19 pandemic in europe: an expert consultation. *Lancet Reg. Health Eur.* **8**, 100185 (2021). 34345876 10.1016/j.lanepe.2021.100185 PMC8321710

[7] Johns Hopkins University & Medicine. Johns Hopkins Coronavirus Resource Center. https://coronavirus.jhu.edu (2022).

[8] Brooks, S. K. et al. The impact of unplanned school closure on children’s social contact: rapid evidence review. *Euro. Surveill*. **25**, 2000188 (2020). 32265006 10.2807/1560-7917.ES.2020.25.13.2000188 PMC7140596

[9] Hoekstra, P. J. Suicidality in children and adolescents: lessons to be learned from the COVID-19 crisis. *Eur. Child. Adolesc. Psychiatry*. **29**, 737–738 (2020). 32488455 10.1007/s00787-020-01570-z PMC7266412

[10] Isumi, A., Doi, S., Yamaoka, Y., Takahashi, K. & Fujiwara, T. Do suicide rates in children and adolescents change during school closure in japan?? The acute effect of the first wave of COVID-19 pandemic on child and adolescent mental health. *Child. Abuse Negl.* **110**, 104680 (2020). 32847679 10.1016/j.chiabu.2020.104680 PMC7443207

[11] Brahmbhatt, K. et al. Adaptations made to pediatric consultation-liaison psychiatry service delivery during the early months of the COVID-19 pandemic: a North American multisite survey. *J. Acad. Consult Liaison Psychiatry*. **62**, 511–521 (2021). 34033972 10.1016/j.jaclp.2021.05.003 PMC8141785

[12] Lloyd-Richardson, E. E., Perrine, N., Dierker, L. & Kelley, M. L. Characteristics and functions of non-suicidal self-injury in a community sample of adolescents. *Psychol. Med.* **37**, 1183–1192 (2007). 17349105 10.1017/S003329170700027X PMC2538378

[13] Leeb, R. T. et al. Mental health-related emergency department visits among children aged < 18 years during the COVID-19 pandemic—United states, January 1–October 17, 2020. *MMWR Morb Mortal. Wkly. Rep.* **69**, 1675–1680 (2020). 33180751 10.15585/mmwr.mm6945a3 PMC7660659

[14] Özek Erkuran, H. & Önen, Ö. Evaluation of psychiatric consultations requested for ınpatient children and adolescents in a training and research hospital. *Turk. J. Child. Adolesc. Ment Health*. **29**, 204–209 (2022).

[15] Kuo, E. et al. Coronavirus disease 2019 (COVID-19) pandemic’s effect on child and adolescent mental health: analysis of pediatric intensive care unit and consultation-liaison psychiatry service. *OBM Neurobiol.* **7**, 1–12 (2023).

[16] Kose, S. et al. Effects of a pandemic on child and adolescent psychiatry emergency admissions: early experiences during the COVID-19 outbreak. *Asian J. Psychiatry*. **61**, 102678 (2021). 10.1016/j.ajp.2021.102678 PMC9760248 34034017

[17] Ravens-Sieberer, U. et al. Quality of life and mental health in children and adolescents during the first year of the COVID-19 pandemic: results of a two-wave nationwide population-based study. *Eur. Child. Adolesc. Psychiatry*. **32**, 575–588 (2021). 34636964 10.1007/s00787-021-01889-1 PMC8506100

[18] Racine, N. et al. Global prevalence of depressive and anxiety symptoms in children and adolescents during COVID-19: a meta-analysis. *JAMA Pediatr.* **175**, 1142–1150. 10.1001/jamapediatrics.2021.2482 (2021). 34369987 10.1001/jamapediatrics.2021.2482 PMC8353576

[19] Ma, L. et al. Prevalence of mental health problems among children and adolescents during the COVID-19 pandemic: A systematic review and meta-analysis. *J. Affect. Disord*. **293**, 78–89 (2021). 34174475 10.1016/j.jad.2021.06.021 PMC9711885

[20] Ougrin, D. et al. Pandemic-related emergency psychiatric presentations for self-harm of children and adolescents in 10 countries (PREP-kids): a retrospective international cohort study. *Eur. Child. Adolesc. Psychiatry*. **31**, 1–13 (2022). 33677628 10.1007/s00787-021-01741-6 PMC7937052

[21] Emiroglu, N., Aras, S., Yalin, S., Dogan, O. & Akay, A. Evaluation of the child and adolescent psychiatric inpatient consultations. *Anatol. J. Psychiatry*. **10**, 217 (2009).

[22] Zhou, S. J. et al. Prevalence and socio-demographic correlates of psychological health problems in Chinese adolescents during the outbreak of COVID-19. *Eur. Child. Adolesc. Psychiatry*. **29**, 749–758 (2020). 32363492 10.1007/s00787-020-01541-4 PMC7196181

[23] Branquinho, C., Kelly, C., Arevalo, L. C., Santos, A. & de Gaspar, M. Hey, we also have something to say: A qualitative study of Portuguese adolescents’ and young people’s experiences under COVID-19. *J. Community Psychol.* **48**, 2740–2752 (2020). 33001455 10.1002/jcop.22453 PMC7537124

[24] Jones, S. E. et al. Mental health, suicidality, and connectedness among high school students during the COVID-19 pandemic — Adolescent behaviors and experiences survey, united states, January–June 2021. *MMWR Suppl.* **71**, 16–21. 10.15585/mmwr.su7103a3 (2022). 35358165 10.15585/mmwr.su7103a3 PMC8979602

[25] Sawyer, M. G. et al. Mental health trajectories from childhood to adolescence: findings from the longitudinal study of Australian children. *Aust N Z. J. Psychiatry*. **56**, 190–200. 10.1177/00048674211029648 (2022).

[26] Gonzalez-Urdiales, P. et al. Pediatric ıntentional self-poisoning evaluated in the emergency department: an international study. *Pediatr. Emerg. Care*. **37**, e1631–e1636. 10.1097/PEC.0000000000002141 (2021). 32541402 10.1097/PEC.0000000000002141

[27] Yıldırım, S., Aşık, A. & Duyu, M. Evaluation of attempted suicide events through oral intake among children in a metropolitan city: a single-center study. *Trends Pediatr.* **5**, 72–78. 10.59213/TP.2024.143 (2024).

[28] Neehall, J. & Beharry, N. Demographic and clinical features of adolescent parasuicides. *West. Indian Med. J.* **43**, 123–126 (1994). 7900374

[29] Gizli Çoban, Ö., Gül, M. & Önder, A. Evaluation of children and adolescents admitted to emergency service with suicide attempt. *J Pediatr. Emerg. Intensive Care Med.* **9**, 153–157 (2022).

[30] Zhang, L. et al. Assessment of mental health of Chinese primary school students before and after school closing and opening during the COVID-19 pandemic. *JAMA Netw. Open.* **3**, e2021482 (2020). 32915233 10.1001/jamanetworkopen.2020.21482 PMC7489803

[31] Chen, F. et al. Depression and anxiety among adolescents during COVID-19: A cross-sectional study. *Brain Behav. Immun.* **88**, 36–38 (2020). 32464156 10.1016/j.bbi.2020.05.061 PMC7247496

[32] Magson, N. R. et al. Risk and protective factors for prospective changes in adolescent mental health during the COVID-19 pandemic. *J. Youth Adolesc.* **50**, 44–57 (2021). 33108542 10.1007/s10964-020-01332-9 PMC7590912

[33] Carballo, J. J. et al. Psychosocial risk factors for suicidality in children and adolescents. *Eur. Child. Adolesc. Psychiatry*. **29**, 759–776 (2020). 30684089 10.1007/s00787-018-01270-9 PMC7305074

[34] Kostev, K., Weber, K., Riedel-Heller, S., von Vultée, C. & Bohlken, J. Increase in depression and anxiety disorder diagnoses during the COVID-19 pandemic in children and adolescents followed in pediatric practices in Germany. *Eur. Child. Adolesc. Psychiatry*. **32**, 873–879. 10.1007/s00787-021-01924-1 (2023). 34825964 10.1007/s00787-021-01924-1 PMC8619647

[35] Pinkse-Schepers, A. L. et al. The development of depression and social anxiety symptoms in adolescents and the negative impact of the COVID-19 pandemic and desire for peer contact. *Front. Public. Health*. **12**, 1374327 (2024). 39354999 10.3389/fpubh.2024.1374327 PMC11442221

[36] Fortuna, L. R., Brown, I. C., Woods, L., Porche, M. V. & G. G., & The impact of COVID-19 on anxiety disorders in youth: coping with stress, worry, and recovering from a pandemic. *Child. Adolesc. Psychiatr Clin. N Am.* **32**, 531–542 (2023). 37201965 10.1016/j.chc.2023.02.002 PMC9894765

[37] Brown, S. M., Doom, J. R., Lechuga-Peña, S., Watamura, S. E. & Koppels, T. Stress and parenting during the global COVID-19 pandemic. *Child. Abuse Negl.* **110**, 104699 (2020). 32859394 10.1016/j.chiabu.2020.104699 PMC7440155

[38] Fegert, J. M., Vitiello, B., Plener, P. L. & Clemens, V. Challenges and burden of the coronavirus 2019 (COVID-19) pandemic for child and adolescent mental health: a narrative review to highlight clinical and research needs in the acute phase and the long return to normality. *Child. Adolesc. Psychiatry Ment Health*. **14**, 20 (2020). 32419840 10.1186/s13034-020-00329-3 PMC7216870

[39] Chung, G., Lanier, P. & Wong, P. Y. J. Mediating effects of parental stress on harsh parenting and parent-child relationship during coronavirus (COVID-19) pandemic in Singapore. *J. Fam Violence*. **37**, 801–812 (2022). 32895601 10.1007/s10896-020-00200-1 PMC7467635

[40] Tang, S., Xiang, M., Cheung, T. & Xiang, Y. T. Mental health and its correlates among children and adolescents during COVID-19 school closure: the importance of parent-child discussion. *J. Affect. Disord*. **279**, 353–360 (2021). 33099049 10.1016/j.jad.2020.10.016 PMC7550131

[41] Sandal Saraç, Ö. et al. Evaluation of child and adolescent psychiatry consultations requested for patients in the pediatric intensive care unit. *Turk. J. Clin. Lab.* **1**, 30–36 (2023).

[42] Sheen, A. et al. 51.7 Impact of Covid-19-related school closures on children and adolescents worldwide: a literature review. *J. Am. Acad. Child. Adolesc. Psychiatry*. **59**, 253 (2020).

